# Supplementary material for: Nanosuspension-Based Dissolvable Microneedle Arrays to Enhance Diclofenac Skin Delivery
Source: Pharmaceutics. 2023 Sep 13;15(9):2308. doi: 10.3390/pharmaceutics15092308 (PMC10536216; doi:10.3390/pharmaceutics15092308)
Supplement: Supplementary file 1 [file pharmaceutics-15-02308-s001.zip › pharmaceutics-2585324-supplementary.pdf]

pharmaceutics

MDPI

Supplementary Material

Nanosuspension-based dissolvable microneedle arrays to enhance diclofenac skin delivery

Luca Casula [1,†], Rosa Pireddu [1,†], Maria Cristina Cardia [1], Elena Pini [2], Donatella Valenti [1], Michele Schlich [1], Chiara Sinico [1], Salvatore Marceddu [3], Nina Dragićević [4], Anna Maria Fadda [1] and Francesco Lai [1*]

[1] Dipartimento di Scienze della Vita e dell'Ambiente, Sezione di Scienze del Farmaco, CNBS, Università degli Studi di Cagliari, 09124 Cagliari, Italy; luca.casula@unica.it (L.C.); rosapireddu@unica.it (R.P.); cardiamr@unica.it (M.C.C.); valenti@unica.it (D.V.); michele.schlich@unica.it (M.S.); sinico@unica.it (C.S.); mfadda@unica.it (A.M.F)
[2] Department of Pharmaceutical Sciences, General and Organic Chemistry Section "Alessandro Marchesini", University of Milan, 20133 Milan, Italy; elena.pini@unimi.it
[3] Istituto di Scienze delle Produzioni Alimentari (ISPA)-CNR, Sez. di Sassari, 07040 Baldinca, Italy; salvatore.marceddu@cnr.it
[4] Department of Pharmacy, Singidunum University, Belgrade, Serbia; ndragicevic@singidunum.ac.rs

* Correspondence: frlai@unica.it; Tel.: +39-070-675-8514
† These authors contributed equally to this work.

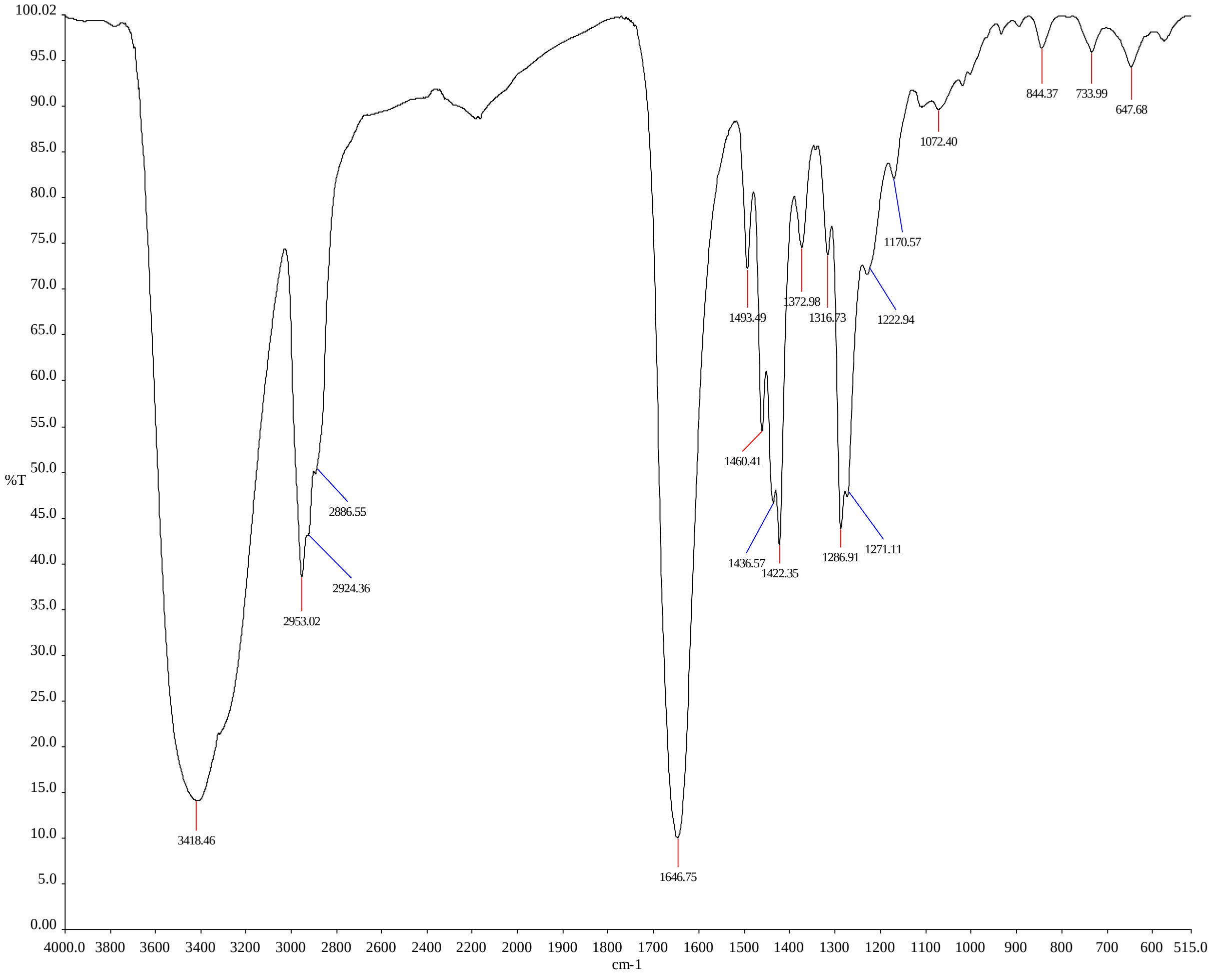

Figure S1. ATR-FTIR spectra of PVP.

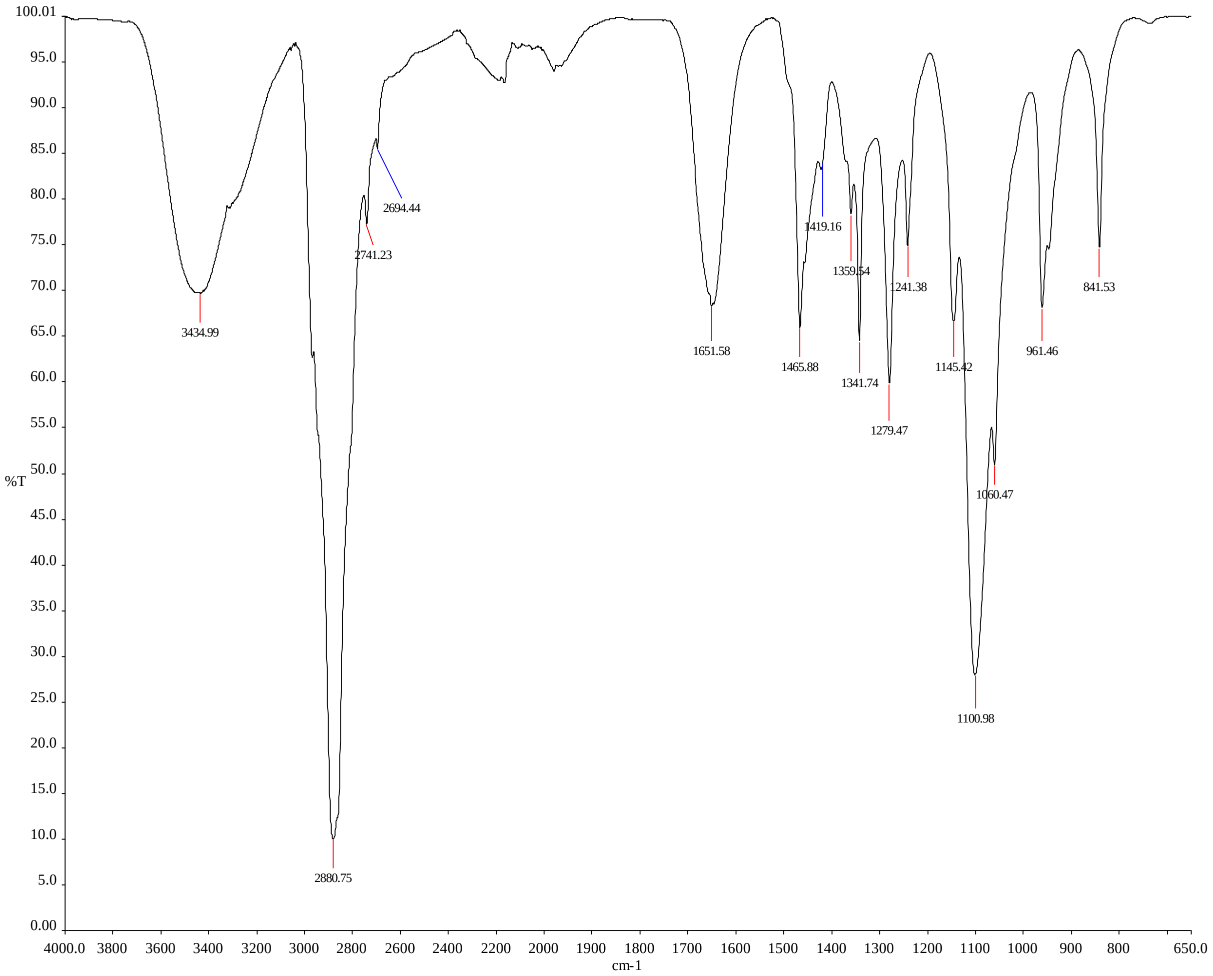

Figure S2. ATR-FTIR spectra of PM PVP/P118 (1:1 w/w).

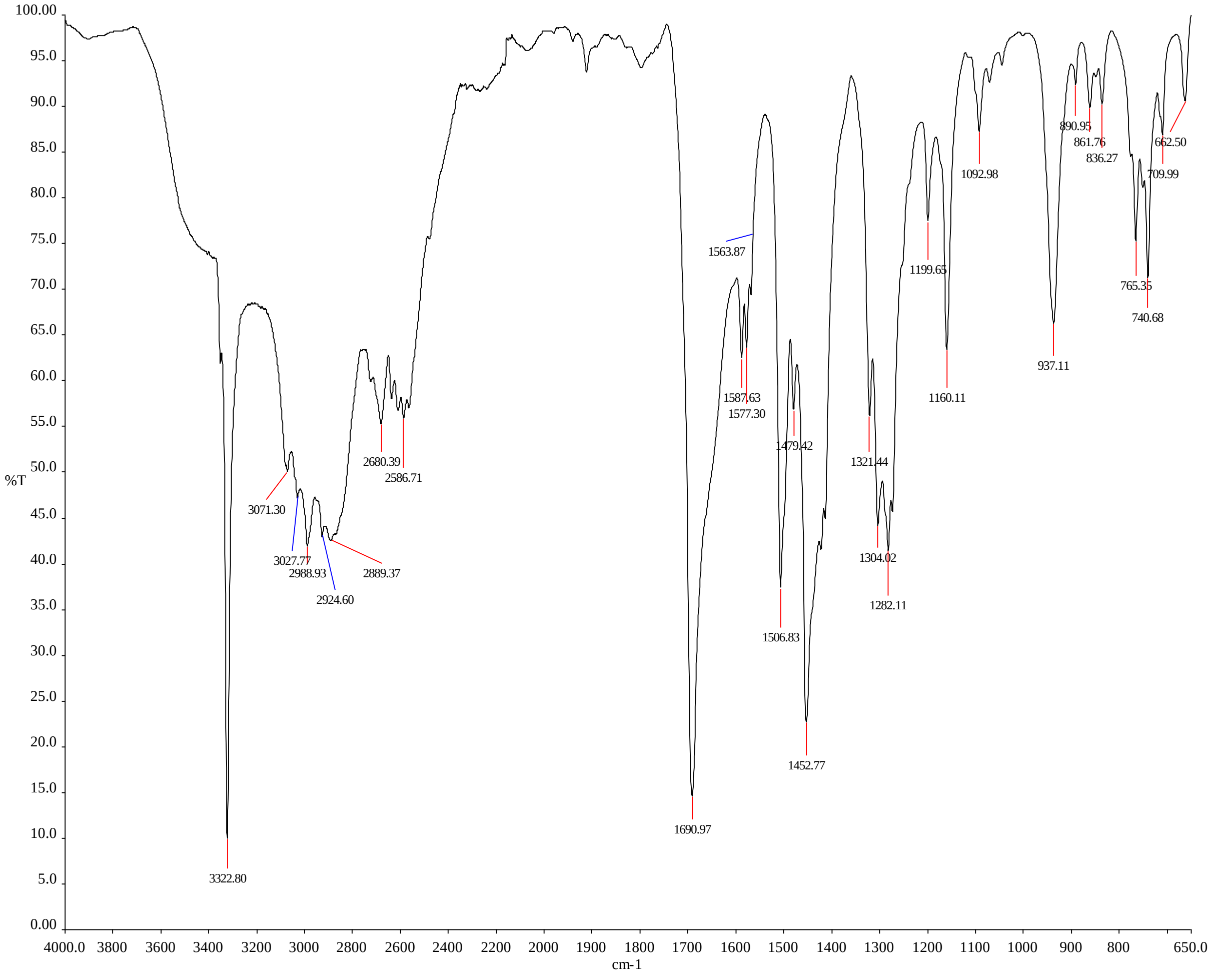

Figure S3. ATR-FTIR spectra of PM PVP/DCF (1:1 w/w).

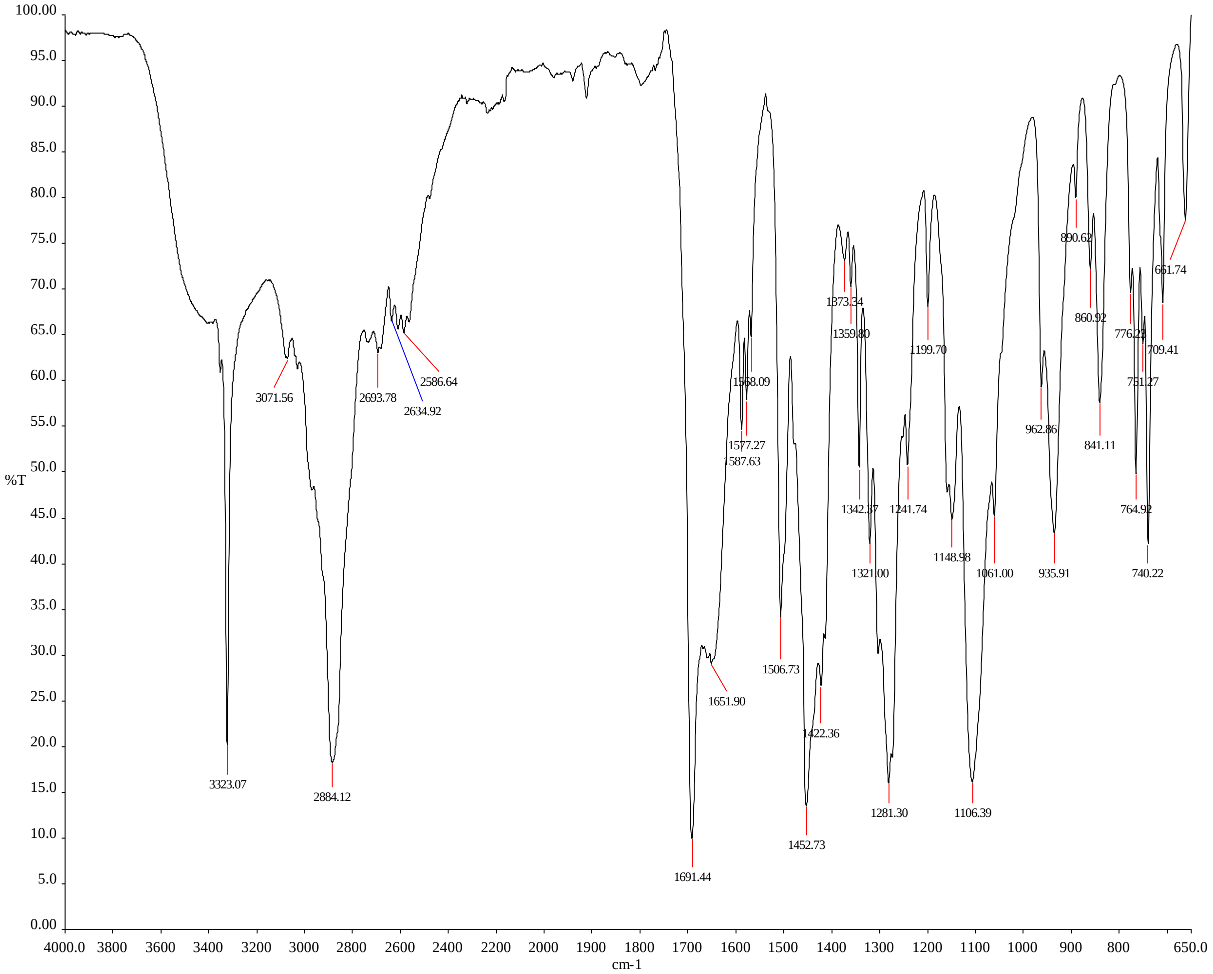

Figure S4. ATR-FTIR spectra of PM PVP/DCF-NS (1:1 w/w).

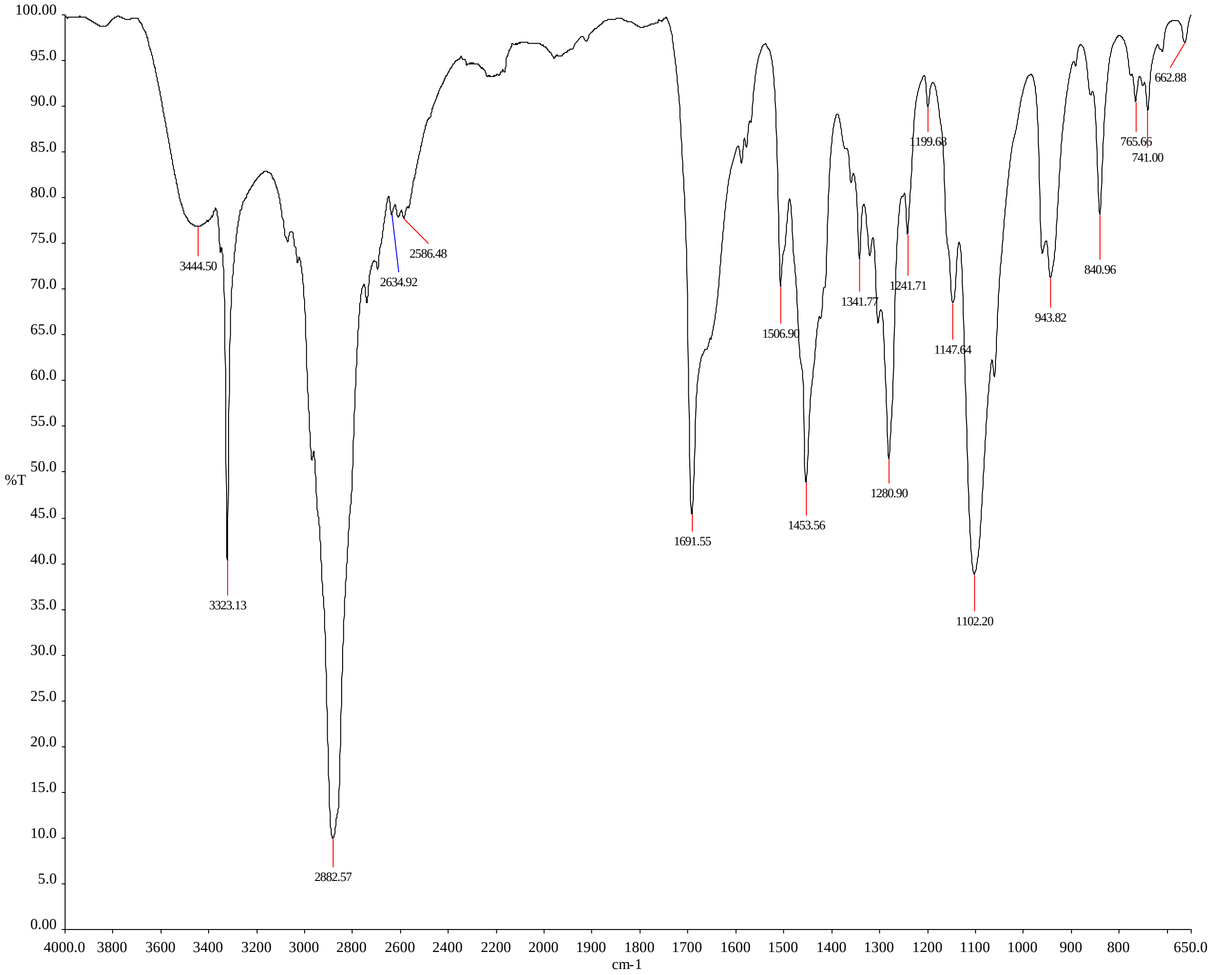

Figure S5. ATR-FTIR spectra of PM PVP/DCF/P188 (1:1 w/w).

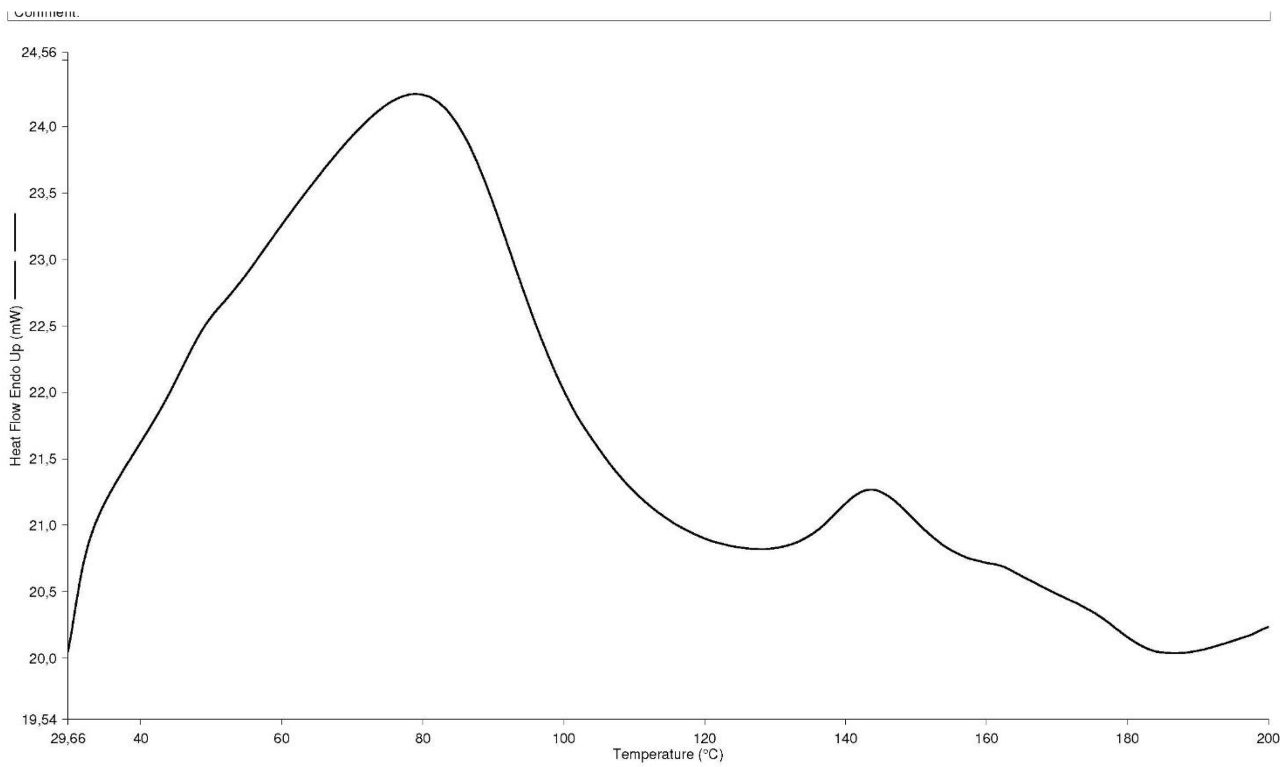

Figure S6. DSC thermogram of PVP K10.

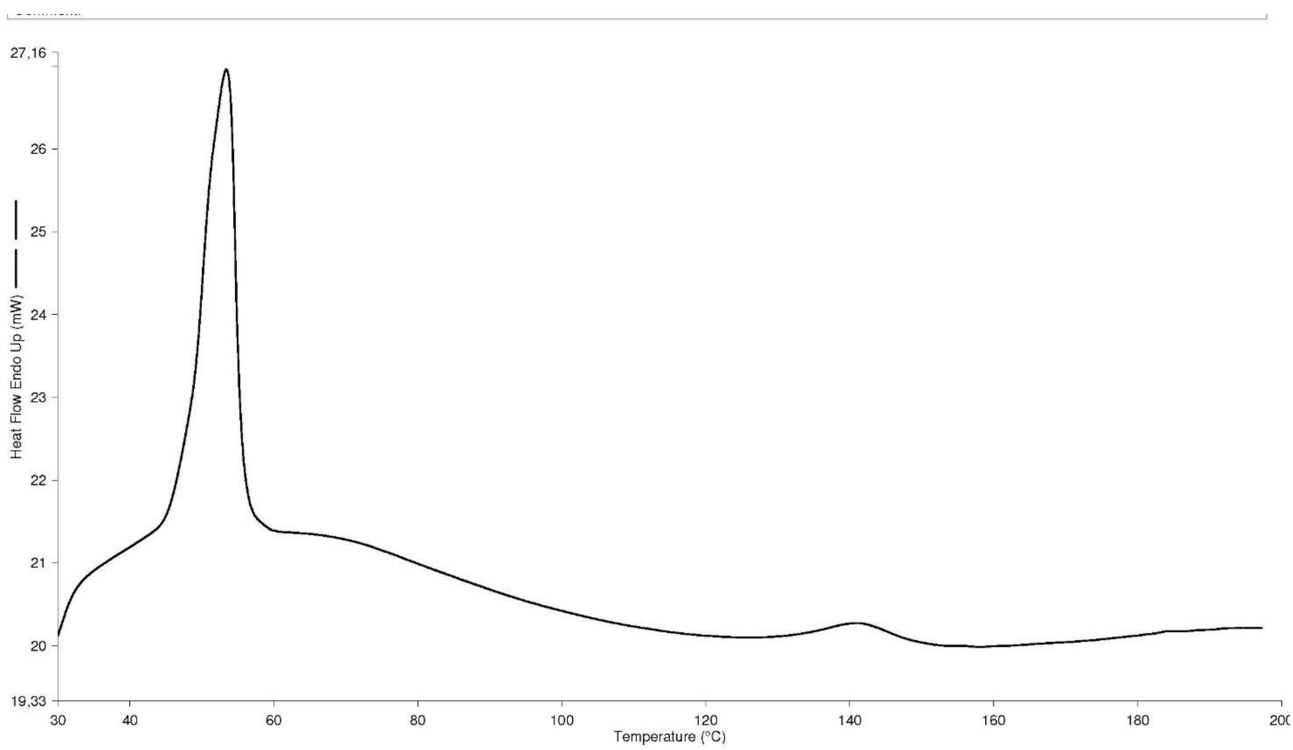

Figure S7. DSC thermogram of PM 1:1 (w/w) PVP/P188.

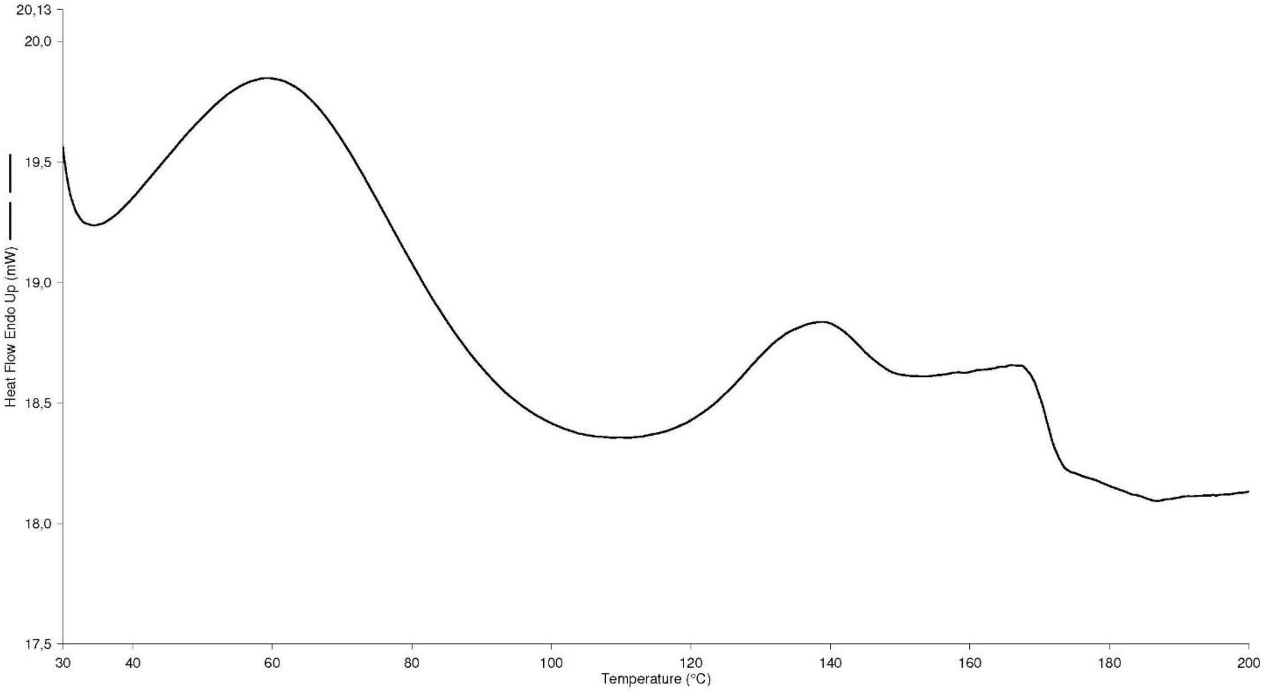

Figure S8. DSC thermogram of PM 1:1 (w/w) DCF/PVP.

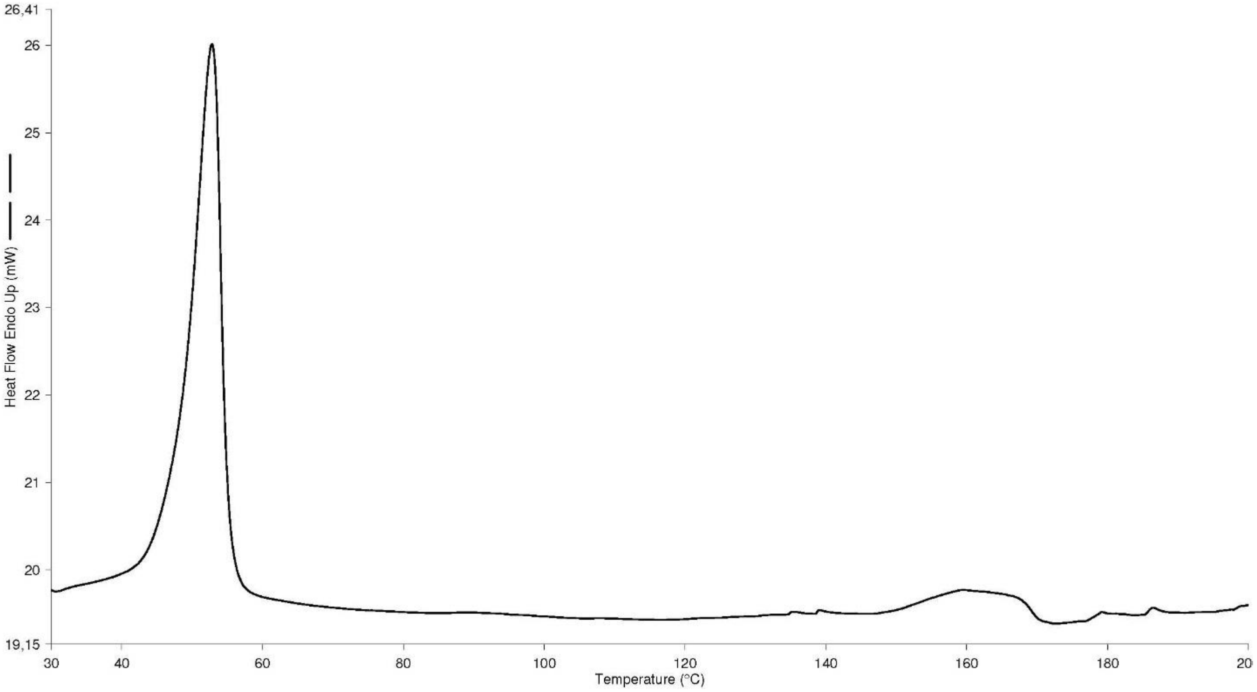

Figure S9. DSC thermogram of PM 1:1:1 (w/w/w) DCF/PVP/P188.

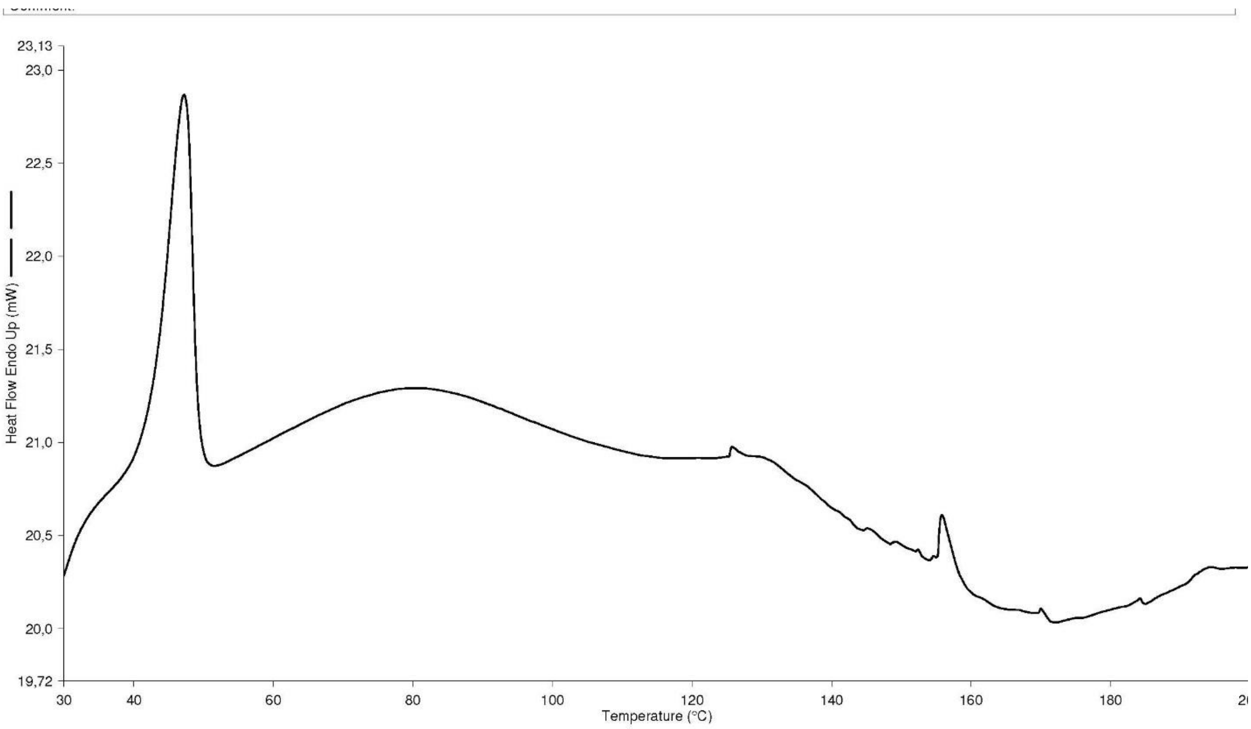

Figure S10. DSC thermogram of PM 1:1 (w/w) NS DCF/PVP.
